# The Journey of Engaging With Web-Based Self-Harm and Suicide Content: Longitudinal Qualitative Study

**DOI:** 10.2196/47699

**Published:** 2024-03-28

**Authors:** Zoë Haime, Laura Kennedy, Lydia Grace, Rachel Cohen, Jane Derges, Lucy Biddle

**Affiliations:** 1 Population Health Sciences Bristol Medical School University of Bristol Bristol United Kingdom; 2 Centre for Society and Mental Health Kings College London United Kingdom; 3 Samaritans Surrey United Kingdom; 4 Department of Health Service and Population Research King’s College London London United Kingdom; 5 Health and Social Services Group Welsh Government Cardiff United Kingdom

**Keywords:** suicide, self-harm, online, longitudinal, qualitative

## Abstract

**Background:**

Self-harm and suicide are major public health concerns worldwide, with attention focused on the web environment as a helpful or harmful influence. Longitudinal research on self-harm and suicide–related internet use is limited, highlighting a paucity of evidence on long-term patterns and effects of engaging with such content.

**Objective:**

This study explores the experiences of people engaging with self-harm or suicide content over a 6-month period.

**Methods:**

This study used qualitative and digital ethnographic methods longitudinally, including one-to-one interviews at 3 time points to explore individual narratives. A trajectory analysis approach involving 4 steps was used to interpret the data.

**Results:**

The findings from 14 participants established the web-based journey of people who engage with self-harm or suicide content. In total, 5 themes were identified: initial interactions with self-harm or suicide content, changes in what self-harm or suicide content people engage with and where, changes in experiences of self-harm or suicide behaviors associated with web-based self-harm or suicide content engagement, the disengagement-reengagement cycle, and future perspectives on web-based self-harm or suicide content engagement. Initial engagements were driven by participants seeking help, often when offline support had been unavailable. Some participants’ exposure to self-harm and suicide content led to their own self-harm and suicide behaviors, with varying patterns of change over time. Notably, disengagement from web-based self-harm and suicide spaces served as a protective measure for all participants, but the pull of familiar content resulted in only brief periods of disconnection. Participants also expressed future intentions to continue returning to these self-harm and suicide web-based spaces, acknowledging the nonlinear nature of their own recovery journey and aiming to support others in the community. Within the themes identified in this study, narratives revealed that participants’ behavior was shaped by cognitive flexibility and rigidity, metacognitive abilities, and digital expertise. Opportunities for behavior change arose during periods of cognitive flexibility prompted by life events, stressors, and shifts in mental health. Participants sought diverse and potentially harmful content during challenging times but moved toward recovery-oriented engagements in positive circumstances. Metacognitive and digital efficacy skills also played a pivotal role in participants’ control of web-based interactions, enabling more effective management of content or platforms or sites that posed potential harms.

**Conclusions:**

This study demonstrated the complexity of web-based interactions, with beneficial and harmful content intertwined. Participants who demonstrated metacognition and digital efficacy had better control over web-based engagements. Some attributed these skills to study processes, including taking part in reflective diaries, showing the potential of upskilling users. This study also highlighted how participants remained vulnerable by engaging with familiar web-based spaces, emphasizing the responsibility of web-based industry leaders to develop tools that empower users to enhance their web-based safety.

## Introduction

### Background

Self-harm and suicide are major global public health concerns, with >700,000 people worldwide dying by suicide each year [1]. Attention has increasingly focused on the role of the web environment in triggering, exacerbating, or normalizing self-harm and suicide [2-4]. The amount of suicide-related information accessible on the web has grown [5], and graphic content depicting self-harm is increasingly available on social networking platforms [6]. Research shows that self-harm and suicide–related internet use is common among young people [7], particularly those who are under psychiatric care [8] and who go on to die by suicide [9].

There is a range of self-focused and social motivations for engaging with web-based self-harm and suicide content. These include accessing ongoing peer support or immediate help during a crisis [2,10,11], documenting recovery from self-harm [1,8], and researching suicide methods [12]. Moreover, research has shown that the ways in which people interact with web-based self-harm and suicide content vary depending on their level of distress [11,13].

The diversity in self-harm and suicide material complicates the experiences of content engagement. Research has identified these content interactions as being both a public health concern and a possible preventative measure [3,14], and studies have recognized the potential for engagement to have both benefits and costs [15]. Content with the potential to harm includes information on high-lethality suicide methods [16], prosuicide websites that may encourage suicide [13], and content describing novel methods of self-harm [17]. Benefits associated with accessing content include the role of the online community in peer support, validation and acceptance of one’s own self-harm or suicide feelings, and the opportunity for altruism when helping others [2,10,18-20]. These benefits may be particularly valuable given existing gaps in mental health care services and the widespread stigma that people who self-harm or experience suicidal thoughts encounter offline [2]. However, a recent review suggested that the impact of engaging with particular types of web-based self-harm or suicide–related content varies both between and within individuals, with content that benefits some having negative consequences for others and vice versa [15]. The review also identified only 4 longitudinal studies on the impact of self-harm and suicide–related internet use. Of these studies, 2 identified preventative effects of suicide prevention websites and web-based health forums on suicidal ideation [21,22]. One study showed minimal effects of search engine helpline notices on future suicide queries [23], and another study found that exposure to self-harm on Instagram predicted suicidal ideation and self-harm–related outcomes [17]. However, none of these studies used qualitative methods with their participants, emphasizing the current paucity of evidence on how self-harm and suicide–related web-based behavior evolves and the long-term effects and experiences of engaging with such content from the user’s perspective, including whether these are brief or permanent.

### Objectives

The aim of this study was to explore the motivations for and consequences of viewing, searching for, and posting web-based self-harm or suicide content over a longitudinal period. Specifically, this study builds on existing knowledge by using qualitative and digital ethnographic methods to explore individual narratives of web-based engagement. Exploration of “significant moments” and points of transition within the web journey could also have substantial implications for the prevention of suicide and reduction of self-harm [24].

## Methods

### Design

This was a 6-month qualitative ethnographic study that investigated the stability and change in engagement with web-based self-harm and suicide content. This involved 3 one-to-one interviews and daily diary completion by participants over the study duration. We selected a 6-month time frame to ensure that we could observe changes over time in web-based engagement and associated behaviors [25,26] while also remaining mindful of the considerable commitment required for this ethnographic approach to maintain retention of participants.

### Ethical Considerations

Ethics approval was obtained from the University of Bristol Faculty of Health Sciences ethics committee (reference: 117491). All participants provided written informed consent before participation, and were informed that they could withdraw from the study (including data withdrawal up to the time of analysis), without giving a reason. During consent, participants were assigned a participant ID used to identify their data and ensure anonymity. They were also informed that their data would be held confidentially and securely by the University of Bristol according to its duties and obligations under GDPR and the Data Protection Act. All participants were also compensated for their time, receiving a total of up to £75 (US $94.79) for full study completion.

### Sampling and Recruitment

UK residents aged ≥16 years who were able to communicate in English and had experience engaging with web-based self-harm or suicide content were eligible. This included posting images, videos, memes, forum posts, blog posts, recovery posts, or comments related to self-harm or suicide or engaging with others’ self-harm or suicide–related content through reposting and reblogging, quoting, liking, sharing, saving, subscribing to, or commenting. They did not need to have previous experience with self-harm or suicidal thoughts or behaviors.

Potential participants responded to advertisements posted between November 2021 and April 2022 on social media platforms (Facebook, Twitter [subsequently rebranded as X], and Reddit subreddits [“r/AdultSelfHarm,” “r/StopSelfHarm,” “r/BPD,” “r/MentalHealthUK,” and “r/malementalhealth”]), via Tellmi—a UK-based young person mental health app), and through charity websites and newsletters (Samaritans, SMaRteN, The McPin Foundation, and MQ Mental Health Research). Advertisements were posted once to platforms or sites until the end of recruitment in April 2022; however, due to web-based posting and reposting, it is possible that they were also shared elsewhere by others. Permission was sought from moderators or administrators before posting. Advertisements included a link to an expression of interest form in which participants consented via completion to the collection of brief demographic information, if and when the person last self-harmed, the way they were engaging with web-based self-harm or suicide content, and what platforms they used. All respondents had engaged with web-based self-harm or suicide content in some way.

This information was used to sample a diverse range of participants from those who expressed interest and target recruitment advertisements. Potential participants were sent the study information sheet via email, and those who were still interested in participating completed a consent form. Interviews were then arranged via email. The demographic data of those who did not participate were deleted. Once 14 baseline interviews had been conducted, the study team considered that there was good participant diversity in ethnicity and sufficient gender diversity. In addition, we had a broad range of platforms and apps represented in participant use. The authors also identified high-quality dialogue data sufficient for analysis and consistent themes to address the research aims. This resulted in the data achieving good information power [27], and therefore, recruitment was terminated. Information power was used as an alternative to data saturation in this study as the diverse nature of participant narratives meant that we were unlikely to reach a point of saturation.

### Data Collection

Written consent to participate was provided by participants before entering the study. Participants were also required to complete a mandatory safety plan, including contact details for someone who could support them, their general practitioner’s details (in case serious safety concerns arose), and a self-care plan that was individually designed by each participant to suit their needs (Multimedia Appendix 1). Study information was sent to the parents or guardians of those aged 16 to 18 years as a transparency measure. However, formal parental or guardian consent was not deemed a requirement by the ethics committee given the ages of the participants involved. As part of the study, a distress protocol was developed with a clinician to manage the risk of worsening mental health or increased self-harm or suicidal thoughts as a result of participation in the study. According to the protocol, participants would first be referred to their own safety plan if their mental health declined as a result of the study. A hierarchy of responses was specified in cases of more serious distress, including the options of offering follow-up support from UK suicide charity “Samaritans” or calling upon the advice of a named senior clinician. However, study-induced distress was not reported by participants during the study, and therefore, such responses were not actioned by researchers.

One-to-one interviews were conducted at baseline and the 3- and 6-month time points via Zoom (Zoom Video Communications) with just the researcher and participant present. The interviews were open-ended and flexible, using probing techniques where appropriate, and structured loosely using a topic guide. The main topics explored were “history of self-harm and suicide feelings”; “current and historic web-based activity related to self-harm and suicide content”; “patterns, motivations, and impact of web-based content engagement”; “critical moments in the web-based content engagement journey”; “keeping safe on the web”; and “experiences of web-based moderation and blocking.” The topic guides were originally refined using feedback from 2 lived-experience experts. Throughout the study, the topic guides continued to be iteratively adapted between interviews, grounding question modifications in the study data. The interviews were conducted by ZH, LK, or LB and lasted between 35 and 80 minutes (with baseline interviews averaging 65 [SD 8.55] min and follow-up interviews averaging 45 [SD 2.87] min). They were audio recorded using an encrypted device and then transcribed.

### Diaries

Participants completed daily diaries independently between interviews. These diaries served as an ethnographic tool and were introduced at the end of the baseline interview. Blank digital templates were then provided periodically via email. Each covered a 4-week period and had 3 main components (daily recording of content engagement, mood ratings, and a weekly reflection of content impact). Each participant was asked to complete 5 diaries in total. Entries were used to formulate personalized follow-up interview schedules in which further information or clarifications could be sought from participants.

### Measures

Self-reported mental well-being data were collected from participants at baseline and monthly intervals to coincide with diary data collection. This was done via surveys on SurveyMonkey and included validated measures for assessing anxiety, depression, and psychological well-being (Multimedia Appendix 2 [28-31]). These data were used to characterize the sample and identify whether changes in mental health and mood reported by participants during the study interviews and in the diary data were reflected in outcome measure scores.

### Data Analysis

#### Descriptive Analysis

Participant baseline demographic characteristics were reported as proportions or frequencies, as appropriate. Individual trajectories for well-being measures were represented visually using line graphs.

#### Qualitative Analysis

A trajectory analysis approach [32] was undertaken to interpret interview data temporally using the following steps:

1. Baseline interviews were transcribed, and then, through coding, themes were derived deductively from topic guide questions and inductively from the data themselves. ZH, LK, and LB separately listed preliminary themes and then refined and revised them collaboratively (Table S1 in Multimedia Appendix 2).
2. Initial matrices were produced for each participant, which included data from the baseline and the 3- and 6-month interviews. These were ordered so that each row was dedicated to a theme established in the previous step. Time points were then assigned to each column. Web-based engagement time points included “initiation,” “historic,” “current,” “never,” and periods of “disengagement and reengagement.” These time points were adapted from the original trajectory approach [31] to preserve the “chronological flow” of the data collected during this study. This allowed us to acknowledge historical content engagement and the nonlinear flow of participant journeys as the levels of engagement fluctuated, ceased, and restarted. This also enabled the inclusion of participants who were only interviewed at baseline (due to dropout) as their data included information about past experiences. Data were formatted according to a “key” using text color to denote the site or platform used and highlighting whether it was related to a significant web event. An event was deemed to be “significant” if the participant recalled it as such or if the researchers found evidence within the narrative that it had a significant impact on the participant’s thinking or behavior. Matrices were developed by extracting relevant quotes or context summaries for 2 participants by ZH, LK, and LB, and once consistency in interpretation was achieved, ZH and LK separately constructed the remaining initial matrices, with ongoing discussion between the researchers to ensure that all the data were captured.
3. Second matrices were then constructed for each participant. These were ordered with the initial themes as column headings. Each row represented an web-based platform or site used by the individual and included condensed versions of the “journey” that participants had experienced for each theme. The comparison allowed us to explore possible patterns in theme content by platforms or sites used. Second matrices were created by ZH for each participant and reviewed by LK and LB.
4. With all matrices complete, ZH, LK, and LB met to discuss similarities and differences across participant trajectories, noting trends, patterns, and outliers. Member checking of transcripts did not occur in this study due to funding and time constraints. During qualitative meeting discussions, overarching longitudinal themes were finalized.

## Results

### Participant Flow

**Figure 1:**
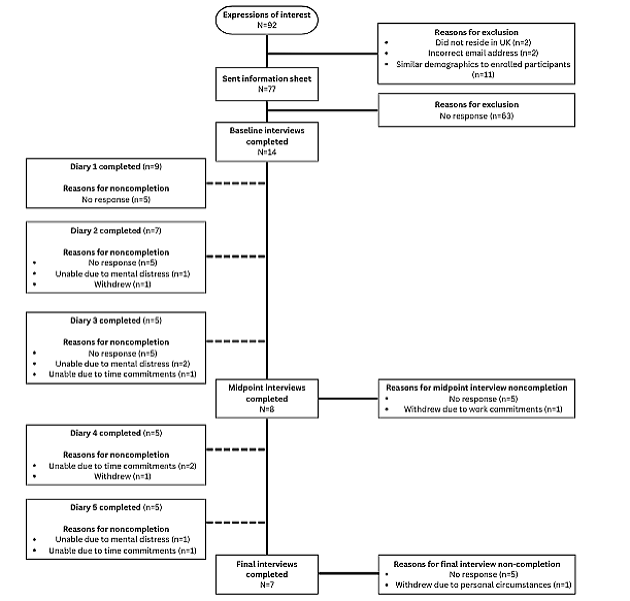
Participant flow through the study. Note: diaries were considered completed if one or more responses were provided.

The participant flow through the study is shown in Figure 1. There were 92 expressions of interest. Of the 77 individuals who were sampled and sent study information, 63 (82%) did not respond and 14 (18%) took part. Data from the expression of interest forms showed that participants were less likely to respond to the research invite if they were younger (aged 16-24 years), had never hurt themselves on purpose, or had self-harmed in the last week.

Of the 14 participants who completed a baseline interview, 8 (57%) completed a midpoint interview, and 7 (50%) also completed the end-point interview. On the basis of preliminary observations of demographic characteristic data from the final sample, it appears that participants of non-British ethnicity may have had a lower likelihood of completing the study compared with those of British ethnicity. However, it should be noted that this observation was not tested statistically (Table S2 in Multimedia Appendix 2). Throughout the study, participants regularly completed their diaries, with study completers returning 77% (27/35) of the distributed diaries.

### Participant Characteristics

The characteristics of those who completed the baseline interviews (N=14) are displayed in Table 1. Of the 14 participants, 4 (29%) self-identified as male and 10 (71%) self-identified as female. Their ages ranged from 16 to 52 years, with 18 to 24 years being the most prevalent age group represented. There was a range of ethnicities, with almost half (6/14, 43%) of the participants being from global majority groups. Participants had engaged with self-harm or suicide–related content on a wide variety of sites and platforms.

**Table 1:** Participant characteristics at baseline (N=14).

Demographic variables			Participants, n (%)
**Gender**			
	Man	4 (29)	
	Woman	10 (71)	
	Nonbinary	0 (0)	
**Ethnicity**			
	Asian British	2 (14)	
	Asian other	2 (14)	
	Black British	1 (7)	
	Black other	0 (0)	
	White British	7 (50)	
	White other	1 (7)	
	Mixed	1 (7)	
**Age (y)**			
	16-17	1 (7)	
	18-24	7 (50)	
	25-35	0 (0)	
	36-45	4 (29)	
	46-54	2 (14)	
	≥55	0 (0)	
**Have you ever hurt yourself on purpose?**			
	Yes	14 (100)	
	No	0 (0)	
**Website or platform used to access content^a^**			
	Instagram	3 (21)	
	Facebook	5 (36)	
	TikTok	1 (7)	
	Twitter	6 (43)	
	Tumblr	3 (21)	
	Weibo	1 (7)	
	Discord	1 (7)	
	WhatsApp	1 (7)	
	YouTube	3 (21)	
	Suicide forums	3 (21)	

^a^Participants were able to select more than one option.

### Participant IDs

Participant IDs were assigned during the consent process to ensure anonymity. As participants were aware of their assigned IDs, these were changed in the manuscript (see further details in Multimedia Appendix 2).

### Descriptive Analysis Results

Individual line graphs for each well-being measure demonstrated fluctuations in mental health throughout the 6-month study period that reflected participant journeys recalled through interviews (Multimedia Appendix 2). One participant, IDB, scored poorer at 6 months on the Entrapment Scale–Short Form (which measures feelings of entrapment in a concise manner) than at baseline; however, the decline was minimal. All other study participants (13/14, 93%) improved from baseline or had no change in total score at the study end point in all quantitative measures, although no statistical analysis of change was undertaken.

### Longitudinal Qualitative Analysis

#### Overview

The themes developed following trajectory analysis included (1) initial engagements with web-based self-harm or suicide content, (2) changes in what self-harm or suicide content people engage with and where, (3) changes in self-harm or suicide behaviors associated with web-based self-harm or suicide content engagement, (4) the disengagement-reengagement cycle, and (5) future perspectives on self-harm and suicide content engagement. The themes and their constituent subthemes are summarized in Textbox 1.

Within these themes, fluctuations in mental health and control were identified as significant factors impacting behavioral and emotional responses to web-based content and, therefore, will be further explored in the following sections.

**Themes and subthemes.**

**Initial engagement with web-based self-harm or suicide content**

- Motivations for initial web-based self-harm or suicide content engagement
- Experience of engaging with self-harm or suicide content for the first time

**Changes in what self-harm or suicide content people engage with and where**

- Changes in types of web-based self-harm or suicide content engagement over time
- Balancing curiosity and control
- Changes in posting web-based self-harm or suicide content over time

**Changes in self-harm or suicide behaviors associated with web-based self-harm or suicide content engagement**

- Personal risk associated with web-based self-harm or suicide content engagement
- The precipitative and protective effects of engagement with self-harm or suicide content on self-harm or suicide behavior

**The disengagement-reengagement cycle**

- Disengagement from web-based self-harm and suicide content
- Reengagement with web-based self-harm and suicide content
- Longer periods of disengagement
- Limiting content engagement: strategies

**Future perspectives on self-harm and suicide content engagement**

#### Initial Engagement With Web-Based Self-Harm or Suicide Content

##### Motivations for Initial Web-Based Self-Harm or Suicide Content Engagement

Our first theme captured historical accounts of engaging with web-based self-harm and suicide content. Participants in this study, most of whom (12/14, 86%) had already self-harmed, initially engaged with web-based self-harm or suicide content following attempts to seek help offline during mental health declines. Those who attended mental health services and received new or changes in diagnoses generally reported leaving unsatisfied, citing reasons that included lack of support, inadequate availability, or feelings of being “dismissed” (IDH; baseline interview) due to a perception of low risk. Some were unable to access services at all or felt that attending was not worthwhile. These mental health declines alongside gaps in service provision were the common catalysts for initial web-based searches for self-harm and suicide content. While some of these searches were motivated by a desire to seek help, they varied among participants, with some also seeking information on self-harm and suicide methods:

So, I’d been to the doctors...I’d already tried looking for help, I was waiting for a referral to the CMHT [Community Mental Health Team]...And then within a couple of days I’d started lightly [cutting] on my hand then I moved up to my arm, and then I was looking for support groups online, just general support groups.

IDB; baseline interview

##### Experience of Engaging With Self-Harm or Suicide Content for the First Time

The experience of initially encountering self-harm or suicide content on the web is captured through the participant responses in Table 2. Only 14% (2/14) of the participants recalled first coming across content unintentionally, with most (12/14, 86%) describing purposeful searches to access material. While most of these searches were for help and support, 14% (2/14) of the participants reported seeking information about methods for self-harm or suicide, and 7% (1/14) of the participants were uncertain about what they were hoping to find but acknowledged that support-focused sites were unhelpful to them at that point.

**Table 2:** Quotations related to the experience of first encountering web-based self-harm or suicide content.

Reaction	Description	Quotations
Negative	First engagement with web-based self-harm or suicide content produced a negative response.	“That’s not what I was looking for [support sites], I didn’t want help, at that point I was beyond help.” [IDH; baseline interview] “...I was researching [a suicide method]...what’s required and the best way to manage [that]...It was scary. It’d have been really easy just to have thought, well, actually, I know more about it now and I can do that.” [IDC; baseline interview] “I received a picture on WhatsApp of someone, of a friend at the time who was self-harming and she basically just sent me a picture of her scars. I think that that image has stayed with me until today, and I think it’s one of the reasons why I’m so careful because it’s not something that I want to see again.” [IDI; baseline interview]
Mixed	Participants experienced both positive and negative responses to the first engagement with web-based self-harm or suicide content.	“...because people were experiencing very similar things to what you were experiencing you wanted to have more of that. It was a good environment in one respect, but it was a very toxic environment in the next because you were listening and you were going, ‘Oh, I’ve been through that.’ But it wasn’t helping. It was actually pushing you down a bit because you were getting ideas [about how to self-harm].” [IDG; baseline interview] “I think I was just surprised that there was so much content out there. And yeah, that they haven’t been removed, and I think...I guess a sort of comfort knowing that there were others out there who were also going through tough times...And I think, I guess also shocked at how severe some [images of self-harm] are yeah.” [IDL; baseline interview]
Positive	First engagement with web-based self-harm or suicide content produced a positive response.	“I applied to go onto that [Facebook] group just so that I could reach out to people and find out more from survivor-led experiences. And people offered support to each other, and I felt that was quite a good thing to do.” [IDA; midpoint interview] “It made me feel a lot less alone just knowing, even if they were anonymous people out on the internet that could be wherever in the world, that there were other people, and I wasn’t the only person feeling like this. It was so beneficial, especially as a young teen.” [IDF; baseline interview]

Some participants sought support-related content, and others not intending to access self-harm or suicide content at all unintentionally came across graphic content (eg, images of fresh self-harm) or suicide method descriptions during their first engagements. Those whose initial interactions were with this type of self-harm or suicide content described feelings of distress even when this was the content they were seeking out. Some of these participants (2/14, 14%) recognized that this content could inadvertently validate and trigger their own self-harm and suicide feelings and behaviors, making them feel more at risk. In cases in which participants first engaged with web-based self-harm or suicide content in a discussion forum or peer support group, they were more likely to respond positively, describing how they felt less alone and were able to share experiences with others. However, some participants (2/14, 14%) had mixed emotions—it was comforting to know similar others existed, but processing extreme content was challenging and subjected them to information about novel self-harm and suicide methods, revealing their lack of control over what they were exposed to.

#### Changes in What Self-Harm or Suicide Content People Engage With and Where

##### Changes in Types of Web-Based Self-Harm or Suicide Content Engagement Over Time

All participants continued interacting with online self-harm or suicide content after their initial encounter even if it had been a negative experience. In cases in which they had positive initial engagements, participants continued to use the same platforms to access self-harm or suicide content in the long term. When those platforms or sites became obsolete, they sought out equivalent content in other web locations. Participants who had negative initial interactions accessed different platforms or sites searching for self-harm or suicide material that resonated with them.

Although participants had self-harm and suicide content that they accessed in a stable and routine manner, many also described occasions when they would change what they were accessing. Most participants (12/14, 86%) explained that different content satisfied different needs depending on their current mental state or mood. Examples of this can be found in Textbox 2.

**Web-based self-harm and suicide content accessed during mental health changes.**

**Change in content accessed due to mental health declines**

- “On a good day I can be in there and I can be supporting others and helping them and building them up. And then on a bad day I’ll be the one looking for support and asking for somebody to you know pick me up a little bit. So, it very much depends on what mood I’m in that day to be honest.” [IDB; baseline interview]
- “If you are depressed and you start like looking at videos that are to do with that sort of thing it’s so easy for you to be in the spiral of just like looking at more and more content about suicide and stuff like that...” [IDK; baseline interview]

**Change in content accessed due to suicidal feelings or intentions**

- “When I am thinking about self-harm, I will just look it up online. I go to the text service when I have suicidal feelings.” [IDF; baseline interview]
- “That’s when [‘if I’m in a really bad crisis’] I’m more seeking it out, so Tik Tok I’m not actively seeking out that content [ok] but that’s when I’m actually seeking it out, thinking I want to die, that’s when I start accessing suicide forums and stuff.” [IDK; baseline interview]

**Change in content accessed due to mental health improvements**

- “I’ve reached a place where I feel like I want to kind of, hear more about recovery and things like that. I think that’s why I found this sort of [‘recovery-based images’] content useful to look at. And I think that, I don’t think I’m triggered by it, but I also don’t want to interact with that kind of content where people are talking about their own [recovery] journeys because I’m not in that kind of place or not in a place where I want to hear about that kind of stuff at the moment. So, yeah, kind of like more interactions with the positive stuff, I think.” [IDI; final interview]

Dips in mental health often resulted in changes in the way participants engaged on the web, such as posting their own self-harm or suicide material rather than just interacting with others’ content. In cases in which participants experienced sustained episodes of poorer mental health, self-harm and suicide content was also seemingly accessed more frequently and sometimes uncontrollably through “habit” (IDG) or “addiction” (IDC and IDH), with 21% (3/14) of the participants describing it as falling down a “rabbit hole” (IDJ, IDL, and IDF). In total, 7% (1/14) of the participants reported how this compulsive engagement with self-harm or suicide content interrupted elements of their usual social and occupational functioning:

Even through work time I would take ten minutes and just read some of it.

IDH; baseline interview

Directly questioning participants about web-based engagement when feeling “actively suicidal” elicited similar reported changes in behavior. A couple of participants described engaging with different content—notably turning to web-based suicide organizations and charities or friends and family members offline when they needed support for suicidal thoughts rather than their usual web-based resources for self-harm or suicide content. However, another 21% (3/14) of the participants described how prominent suicidal thoughts were more likely to result in them returning to prosuicide forums, where they would seek or check resources for their own suicide plans.

Improvements in mental health saw participants more likely to transition to web content of a recovery-based nature while often sticking to the same web-based locations. Some participants (3/14, 21%) also attempted to limit web engagement with greater use of offline resources such as community help centers or taking part in meaningful activities.

##### Balancing Curiosity and Control

Other participants came across content unexpectedly in their web journey or seemed to spontaneously seek out different self-harm or suicide content due to “curiosity.” Some described the ability to negotiate novel self-harm and suicide content with a developed sense of control over time, skipping over or avoiding engaging with content that was undesirable to them:

Being able to scroll past content with trigger warnings of self-harm pics has been quite a new thing. Like in the last year-ish, before then I wouldn’t have been able to have done that. I’d have looked.

IDJ; baseline interview

However, others described tensions between curiosity and control and how that curiosity led them to seek out different self-harm or suicide content. For example, 14% (2/14) of the participants, who read a news article on a person’s death by suicide that referenced web-based prosuicide forums, went on to search for them:

...I saw it [article on death-by-suicide of person who used pro-suicide forums] in the news. When you see something in the news, especially on the BBC website you know...it’s quite serious stuff. So, then you end up looking further. Now sometimes you have to be careful because you get drawn into it and I think you have to sort of say to yourself, “I’m only going to spend a few minutes doing this...”

IDE; midpoint interview

The functions of social media sites (eg, hashtags or algorithms) could also enable unintentional content encounters, making control over engagements less feasible:

I guess sometimes that like tags on social media and...it’s usually by chance, I don’t actively go and seek them, but sometimes it appears and then I kind of just go down a rabbit hole of looking at more of such content. Even though I didn’t do it intentionally.

IDL; baseline interview

Another participant explained that, in transitioning from self-harm and suicide content that no longer resonated, they had less control over what they engaged with:

I think recently, it’s like I don’t know what I’m looking for, but it’s like I know that I haven’t been able to find it...So, I think it’s normally looking through my explore page instead of searching for anything in particular...

IDI; midpoint interview

##### Changes in Posting Web-Based Self-Harm or Suicide Content Over Time

For some participants (5/14, 36%), posting content seeking help and support regarding self-harm or suicide feelings or looking for ways to stay safe while self-harming was an early action in their web journey (IDA, IDB, IDC, IDD, and IDG). Others also posted detailed descriptions of suicide methods they were considering on discussion forums (IDH, IDK, and IDD), blog posts detailing their own self-harm and suicide feelings (IDN), and images of quotes on Instagram with captions about their mental health (IDI). One participant sent images of their own self-harm via direct messaging after other users requested them (IDK).

A total of 21% (3/14) of the participants in the study refrained from publishing their own content publicly (IDF, IDL, and IDJ). Of these 3 participants, 2 (67%) posted content privately (meaning that it was posted on the web but was only visible to them). Both participants described this as their way to “vent” (IDF) or “rant” (IDL) when upset and an opportunity to document their journey.

Notably, all 3 “observation-only” participants mentioned valuing their anonymity in the web space and refraining from online community interactions. They also emphasized that the potential for posts to negatively affect others deterred them from posting self-harm or suicide content publicly:

I always felt quite conflicted about reposting other people’s content related to it [photos or videos of fresh self-harm]. I feel like it’s one thing for me to look at it because they’ve posted it...versus me reblogging it to my own blog. I don’t know. It’s odd to explain it but it just felt weird.

IDF; baseline interview

Another participant reported posting content in one context (asking for support on a Facebook group) but not posting “graphic images” (IDC) due to fear that it may cause harm to children. This particular concern for young people viewing content was echoed by IDF, IDH, and IDK.

IDN, who initially described making public blog posts about their own self-harm, later made these private due to a realization that the material may negatively affect others as well as an attempt to maintain anonymity. IDI also reported a change in posting behavior during and as a result of taking part in the study. After initially posting about their experiences in an attempt to raise mental health awareness, they reflected on their tendency to put a “positive spin” (IDI) on content, and by the 6-month follow-up interview, they had reduced the frequency of their posts as they began to question their own authenticity. They considered that, if they posted about their negative experiences, it would likely have a harmful effect on others, and so they refrained from posting.

Finally, one participant also noted that access to psychological therapy reduced their need to post on the web for support:

[I haven’t posted] for quite some time actually. I can’t remember the last time I did that. It would be over a month ago easily. Yeah, I haven’t needed to really.

IDA; final interview

Why do you think that is?

Interviewer; final interview

Because I could handle whatever I was thinking probably on my own or bring it to the next...because I’m having weekly sessions with my psychologist...

IDA; final interview

#### Changes in Self-Harm or Suicide Behaviors Associated With Web-Based Self-Harm or Suicide Content Engagement

##### Personal Risk Associated With Web-Based Self-Harm or Suicide Content Engagement

As described previously, some participants identified risks after their initial exposure to web-based self-harm or suicide content (Table 2). Others recognized potentially harmful consequences after longer periods of engagement. Some thought that the content they engaged with gave them implicit “permission” to carry out similar self-harm or suicide behaviors (IDG, IDH, IDJ, IDK, and IDN):

It makes it [completing suicide] feel less scary and like being able to hear people talk about what happened to them, them saying it’s not that bad, like it wasn’t...it just felt like nothing, it makes it feel a lot easier to do it if you know what I mean?

IDK; baseline interview

Some found that their own self-harm or suicidal behaviors were influenced by self-harm and suicide information they had gathered on the web (IDJ, IDK, IDL, and IDB):

...there were some posts which would link to other websites where you could get resources [information on overdose statistics]. I’d say definitely at the start of my mental health journey that was quite a turning point for me. Because it was just an idea and then it became a possible thing to do.

IDJ; baseline interview

Another participant experienced feelings of jealousy over the self-harm people had engaged in, which resulted in them feeling the need to escalate their self-harm behaviors:

I think that was that self-comparison to myself...maybe I’m being too scared or I’m not trying hard enough...

IDL; baseline interview

##### The Precipitative and Protective Effects of Engagement With Self-Harm or Suicide Content on Self-Harm and Suicide Behavior

The feelings and behaviors that participants experienced following engagement with web-based self-harm or suicide content are shown in Table 3. Content could be precipitative or protective for participants depending on when they encountered it in their journey. Several participants (5/14, 36%) recalled engaging in self-harm and suicide behavior as a result of engaging with web-based content. A few of these participants (4/14, 29%) went on to describe changes in their self-harm and suicide behaviors related to content engagement over time, implying that the effect could change from precipitative to protective. This included living vicariously through others’ self-harm images (IDF and IDL), finding content engagement relaxing (IDC), and using content searches as a way to delay or stop their own suicidal behavior (IDD). One participant suggested that such changes in behavior were due to building their own mental resilience over time:

I wouldn’t say the internet content changed, it would be more like I’ve changed to deal with what the internet’s providing me.

IDJ; baseline interview

Another participant recognized the need to consistently “check-in” with their own mental health before engaging on the web:

It’s just about how I’m feeling, like do I feel like I have the capacity to deal with the internet really, do I actually want to look at what people are saying and what people are posting.

IDF; midpoint interview

For some, there was less consistency regarding whether engagements with self-harm and suicide content would result in helpful or harmful circumstances. This was exemplified by one participant who stated that their searches were usually protective and kept them occupied when their suicidal thoughts were most intense:

I think there is a part of me that does it [conducts searches for self-harm and suicide content] to buy time.

IDB; final interview

However, this participant also reported attempting a new form of self-harm at the midpoint interview after learning about it through a peer support group on Facebook.

**Table 3:** Precipitative and protective effects of web-based self-harm or suicide content engagement identified by participants.

Factor and description			Quotations
**Precipitative factors**			
	Self-harm or suicide behavior as a consequence of engaging with web-based self-harm and suicide content	“It could also be really detrimental because many times, I would just come away feeling much more triggered than previously and then would engage in the behaviour [self-harm].” [IDF; baseline interview] “One of the [posts] got taken out of a group [by me] because it was talking about bloodletting and since then, I’ve bought syringes and needles to try and do it myself.” [IDB; midpoint interview] “How did you then cope with the fallout of what you’d seen [distressing self-harm and suicide content]?” [Interviewer; baseline interview] “I coped by self-harming. Yeah, and I write lots as well. So yeah, writing about how I feel and what I saw.” [IDC; baseline interview]	
**Protective factors**			
	Vicarious experiences through self-harm or suicide content	“It would mainly be trying to vicariously live out things through other people. So, I had a particular urge but wasn’t in a position where I felt like I could self-harm or necessarily wanted to and almost living those experiences through somebody else’s experience which was one of the ways that it [viewing self-harm material] could be really beneficial for me because it could almost meet that urge without me having to engage in the behaviour.” [IDF; baseline interview]	
	Delaying or stopping own self-harm or suicide behavior	“I don’t really need to research it [suicide method] anymore. Sometimes, I do it anyway and I just re-research, re-read it and re-check my facts but it can be a way of preventing me from doing anything.” [IDB; final interview] “How do you mean?” [Interviewer; final interview] “It’s like there are levels to it, aren’t there? That’s what I find anyway. It starts with thoughts, then it turns to urges and once you get to that urge stage, you need to feel like you’re doing something, whereas, researching it [suicide method] is better than actually putting the tablets in your mouth. It gives you that extra step before you get to that point, if you see what I mean.” [IDB; final interview]	
	Calming effect	“How did you feel [coming across images of self-harm]?”[Interviewer; midpoint interview] “Quite relaxed because that’s what I do [self-harm], so I could identify with them, those people who’d done things like that.” [IDC; midpoint interview]	

#### Disengagement-Reengagement Cycle

##### Disengagement From Web-Based Self-Harm and Suicide Content

Most participants (8/14, 57%) reported entering a cycle of disengagement and reengagement during their web-based self-harm and suicide content journey. Disengagement was usually temporary, with participants choosing to have “no phone days,*”* deleting their accounts, finding offline activities to take part in, or being forced to disengage due to lack of internet access.

Most often, disengagement was purposeful but impulsive. It would usually occur during periods of compulsive engagement when participants recognized a lapse in their control or as a reaction to a significant life event that resulted in mental health decline. Life events that occurred during this study included suicide bereavement, hospitalization, bullying or victimization, and experiences of exam- or work-related stress. The act of intentionally disengaging from self-harm or suicide content was usually a conscious decision to reclaim control over their web-based actions.

A total of 14% (2/14) of the participants reflected on changes in their disengagement behavior while in the study (IDC and IDI). Previously, similar to other participants, they reported a tendency to compulsively access content during periods of poorer mental health followed by impulsive disengagement. However, at the 6-month interview, both participants described an improved ability to recognize their patterns of web-based behavior (Table 4). This understanding and insight empowered the participants to purposefully disengage during declining mental health episodes as a strategic means of regaining control over their behavior.

However, when other participants were forced to disengage from content because of intermittent internet access or physical health problems, they were often left with feelings of loss. Although one participant described this unintentional disengagement as an opportunity for brief respite from self-harm and suicide content engagement, its existence remained a reassuring presence:

I knew I could access them if I needed to, but I thought, “No, I’m having a week off and I’m going to try and distance myself from this as much as I can.”

IDA; final interview

**Table 4:** Reasons for disengagement from web-based self-harm or suicide content—from final interviews.

Participant ID and reason for disengagement		Quote
**IDC**		
	Mental well-being	“It felt like I needed to look after myself and that I needed that break to try and keep myself safe. One of the things that this research has taught me and helped me understand, it’s helped me understand more about how social media impacts upon me. So, I think social media can be a source for good. I think you also need to recognise that sometimes you need a break.”
	Regaining control	“I really crashed down, and it scared me because I’d had a lovely weekend. Things are generally a lot better, and it scared me in that I can still crash down and fluster myself. I didn’t trust myself to be researching suicide, self-harm...And there was a part of me that knew that I wanted to live, there was a part of me that knew I could spiral out of control, and I didn’t want to spiral out of control. And I’ve alluded to the fact that I’ve learnt personally a lot about myself during the six month’s research and how I use social media. And for me that Monday when I made that decision [to disengage from self-harm content] it was really positive.”
**IDF**		
	Regaining control	“I think just to prevent myself from falling down a rabbit hole and looking at content that I know wasn’t good for me. And just feeling like so I’ve always been one of those people that I sort of like to sort of physically remove myself from things and remove things from me. So that’s one of the reasons why I do that.”
	Mental well-being	“So, I think it was about a month ago now and someone who was quite active in Twitter (X) and the mental health recovery community passed away from what I feel was suicide. That’s not been confirmed but when all of that happened, I did take a couple of days off the internet just to, I guess, process things there.”

##### Reengagement With Web-Based Self-Harm and Suicide Content

Participants described various reasons for reengagement with web-based self-harm and suicide content, including a “fear of missing out” with the community (IDA, IDC, IDM, and IDK), wanting to use the site or platform to access other types of content (IDE, IDF, and IDI), procrastination or boredom (IDI and IDK), and the need to perform web-based responsibilities (eg, work or moderating roles within self-harm and suicide communities; IDI, IDA, and IDB). Some participants (5/14, 36%) claimed that they weighed the advantages and disadvantages of web-based content engagement before reengaging. Several participants (3/14, 21%) felt that the benefits of reengaging with self-harm and suicide content, such as feeling part of a community, were enough to justify the potential risks. As this participant noted, while the experience could be upsetting at times, it was still considered useful in light of the rewards of engagement:

With Twitter [X], I deleted that as well, but I felt like actually I missed the community and felt out of touch with people, so I actually found that useful [reengaging], as much as sometimes it’s upsetting, it was useful.

IDK; baseline interview

There were also differing accounts of reengagement due to mental health improvements and declines. One participant described feeling more in control once their mental health was stable:

I think I was in a better place emotionally and with my mental health...And I just felt stronger, I genuinely felt stronger and more positive. It’s a better time of year for me...I’ve started some new medication...So, I think that’s a factor as well and me feeling stronger to go back online. I just felt ready.

IDC; final interview

Similarly, another participant felt that they were more able to view and contribute to self-harm and suicide content in a positive way when their mental health improved:

When my mental state is better, and I can go back on. I feel like I can share, and I can help someone.

IDM

Alternatively, some participants (2/14, 14%) described past reengagement with self-harm and suicide content to “punish” (IDF; baseline interview) themselves for thinking about or carrying out self-harm behaviors:

I think at that time I was kind of trying to make myself feel worse, because it was like, “You need to feel more guilty for what you’re doing.”

IDI; final interview

However, these participants described differences in their reengagements over time. IDI reported how their reengagement behavior changed during the study. When feeling low, they now went on the web and sought out non–self-harm or suicide content.

Other participants also described attempts to engage with self-harm or suicide content differently during the reengagement period with the aim of regaining control. This included observing interactions rather than actively participating or limiting engagements with specific content on platforms or sites:

Recently I’ve just been viewing [prosuicide threads] and I’ve got to fight the urges [to interact].

IDH; baseline interview

However, most participants who disengaged briefly would return to their usual use of web-based content. This reengagement process highlighted weaknesses in participants’ ability to exercise control over web-based actions, leaving users vulnerable to reencountering triggering content on the web and beginning the disengagement-reengagement cycle again:

I basically quit Tik Tok for three weeks because I was like I just can’t deal with it anymore because it’s just so hard to block everything and I was also thinking is it actually good for my mental health and it’s not...

IDK; baseline interview

...but you are back on TikTok now, is that right?

Interviewer; baseline interview

I think I was just bored really, and I thought do you know what I’ll just download it for the afternoon, and...

IDK; baseline interview

##### Longer Periods of Disengagement

In total, 14% (2/14) of the participants in this study disengaged for up to a month before reengaging with specific platforms. One of these participants disengaged after a second death by suicide in their Twitter community. Notably, an earlier death by suicide of another member of the same community had increased their frequency of accessing the platform.

During their Twitter disengagement, the participant continued their engagement with a self-harm support group on Facebook, where they felt less connected:

I think because I haven’t known them [Facebook users] so long and there’s certain people [on Twitter, subsequently rebranded as X]...who post frequently, several times possibly in a day...I think the more you get to know people and recognise the handles, I know it sounds bizarre, but you feel yourself becoming closer to them.

IDC; final interview

Despite this, they also reported beginning to reengage with Twitter toward the end of the study:

I think just because I feel a bit better, I wanted to check-in on other things on there on my newsfeed, wall thing.

IDC; final interview

One other participant disengaged twice from a prosuicide forum. First, they described disengaging following an article on the parents of forum members who had died by suicide. The participant reached out to the parents, and the resulting relationship led to their disengagement:

...they’ve told me I need to get off the site.

IDH; baseline interview

However, they reengaged shortly after this event after wanting to check whether “they [the site] put the resources [suicide methods literature] back” (IDH; baseline interview) following their removal after the media article publication.

At the midpoint interview, IDH had again disengaged from and reengaged with the forum following the death by suicide of a relative. On describing their reengagement, they reported that “it was to check [that] the sources of getting stuff [suicide materials]...are still available” (IDH; midpoint interview) as they were aware of scams related to sourcing materials and wanted to verify that their plans would still be viable.

##### Limiting Content Engagement: Strategies

After spending time engaging with web-based self-harm and suicide content, half (7/14, 50%) of the participants began to develop strategies to limit their content engagement. These included less “arbitrary ‘liking’” to curate their feeds (IDI), clearing search histories to “remove temptation” (IDJ), “blocking” or “muting” certain terms or phrases—such as “suicide” and “self-harm” (IDC, IDF, and IDK)—closing their direct messages so that other users were unable to message them (IDI and IDH), “self-banning” so that they were unable to post (IDH), distracting themselves with positive web-based content (IDE, IDI, IDC, and IDF), “starting new accounts” to avoid tailored algorithms (IDK), and distancing themselves from a self-assigned “role” such as being a mental health advocate (IDJ and IDI). In this study, we observed that younger participants predominantly used these strategies, possibly because of the enhanced accessibility to safety features on the platforms or sites they frequented or their proficiency in digital skills. However, it is noteworthy that most participants regardless of age reported learning digital safety methods of limiting engagement over time through their experiences on the web:

As I’ve got older I’ve realised that actually you know what you see online can really impact on you, and that you know no-one’s going to police it for me so I have to be sensible about the types of people that I follow and the types of things that I do online. I think that’s something that came with sort of getting a bit older.

IDF; midpoint interview

#### Future Perspectives on Web-Based Self-Harm and Suicide Content Engagement

When asked, none of the participants reported a desire to disengage from web-based self-harm or suicide content entirely in the future. Many alluded to the nonlinear nature of their engagement, recognizing difficulties during previous attempts to disengage. Some also described a sense of comfort and reassurance knowing that content continued to exist on the web:

It’s a cushion for people who need that.

IDA; midpoint interview

In addition, others reported a desire to “give back” and described having a peer support role themselves as a future goal following their recovery (IDI, IDM, and IDB):

I’m looking forward to where I improve myself, and maybe be able to talk to more people and if possible, reach out to them, and offer that help.

IDM; final interview

I’m also very passionate about sharing stuff I’ve learnt. When people are in that place that I remember being in and you can see it from their posts, I think, “I’ve just learnt about something that will help them. I’ll pass that on to them.” It’s helping to build that confidence back up to do those posts and say those things on there.

IDB; final interview

Some participants in this study (3/14, 21%) also highlighted that they were unable to find alternative web-based or offline spaces that satisfied their current needs. One participant mentioned that disengaging from their current preferred site or platform could be detrimental and so expressed no wish to “move on”:

What I’m trying to say is that there is nowhere for people when they come off that website. There’s no safe space. There’s nowhere. If you’ve been on that particular site [prosuicide forum] for the reason of wanting to die and you didn’t, there’s nowhere. You’ll go on something and just get these silly comments or things where there’s lack of understanding that just escalates a situation.

IDH; midpoint interview

A few participants in this study (3/14, 21%) did recognize the potential costs associated with continuing to engage in web-based spaces with self-harm and suicide content but compromised, stating that “I do feel that the benefits outweigh the risks” (IDC; baseline interview). For these individuals, the draw of the positive aspects of such content was strong enough to justify the potential negative consequences. Other participants (2/14, 14%) struggled to weigh the risks and benefits of engaging with self-harm and suicide content as they felt that the positive and negative aspects of engaging with content were more intertwined, making it difficult to control what they were exposed to:

I’d say that online is very complicated, depending on what you feed your mind, because it has both positive and negative information, so sometimes it’s good to your mind, and sometimes not. Also, if you are coming across lots of negative things in a group, that can be harmful, like self-harm pictures. But it’s also good to look in those groups for people who are offering help for those things, so that you are learning how to help yourself.

IDM; final interview

Ultimately, this resulted in both sets of participants remaining vulnerable to the negative effects of harmful content as they continued to engage with web-based self-harm and suicide material.

## Discussion

This study showed that those engaging with web-based self-harm and suicide content experienced nonlinear journeys that were characterized by 5 key themes: “initial engagements with web-based self-harm or suicide content,” “changes in what self-harm or suicide content people engage with and where,” “changes in self-harm or suicide behaviors associated with web-based self-harm or suicide content engagement,” “the disengagement-reengagement cycle,” and “future perspectives on web-based self-harm and suicide content engagement.”

### Cognitive Flexibility Versus Cognitive Rigidity

Constructs that may explain behavior change and maintenance within these themes are cognitive flexibility and its counterpart, cognitive rigidity [33]. Cognitive flexibility refers to an openness in thinking and behavior, which allows an individual to consider alternative perspectives and approaches. In contrast, cognitive rigidity is the tendency to adhere to specific thought and behavior patterns, making it challenging to change one’s mindset or actions [33]. Previous research has identified a relationship between cognitive rigidity and suicidal ideation [34] and between cognitive rigidity and self-harm [35]. Another study showed that cognitive flexibility can result in engagement in multiple methods of self-harm [36]. This indicates that the construct of cognitive flexibility may provide important insights into the behavior changes over time associated with web-based self-harm and suicide content engagement. This discussion will explore the ways in which cognitive flexibility was impacted by participants’ mental health and control over decision-making and how this influenced their web journeys.

Previous research has identified gaps in clinical support as a key motivator for web-based self-harm and suicide content engagement [2]. The causes for initial engagement in this study were consistent with this, with participants reporting a lack of support but also a reluctance to engage with clinical services due to previous experiences. This suggests a high level of cognitive flexibility among participants during their first engagement with web-based content, with mental distress and a lack of alternative resources potentially triggering participants to be more open to web-based options. This emphasizes the critical need for accessible offline options during the early stages of mental health decline, preventing vulnerable people from resorting to web-based avenues where they may lack the control or knowledge to engage safely.

When participants were unable to find content that was immediately desirable to them, they explored different self-harm or suicide–related material on the web. Often, this led to spontaneous browsing of self-harm and suicide–related links or hashtags, a behavior characterized as “pessimistic browsing” [13]. While this reflects a high level of cognitive flexibility among participants, it also indicates what might be a lack of behavioral control, making participants vulnerable to potentially harmful encounters. Later on in web journeys, when browsing routines had been established, some reported similar bouts of “pessimistic browsing” and harmful behaviors that they considered spontaneous. These episodes of cognitive flexibility were usually triggered by unexpected exposure to web-based self-harm or suicide content, where impulsive tendencies resulted in exploring this novel content further or, in one case, in trying a new self-harm method. This indicates that unexpected engagements with self-harm or suicide content may act as a stimulus for activating cognitive flexibility, resulting in changes in behavior [37]. When experiencing poor control, this cognitive flexibility may lead to a willingness to engage in potentially unhelpful or harmful behaviors when engaging with self-harm or suicide content [38].

Outside of episodes of cognitive flexibility, participants largely accessed web-based self-harm or suicide content in a routine pattern while also reporting a greater feeling of behavioral control. This cognitive rigidity often worked as a coping mechanism allowing for regular engagement with resources of help and support. However, in instances in which content included images or videos of “fresh self-harm,” suicide, or self-harm and suicide method information, repeated engagements were more likely to have negative effects on participant well-being and sometimes led to increased severity of harm to themselves. This shows that, while some perceived their cognitive rigidity as a form of control, it may ultimately have diminished their ability to make decisions to protect themselves and seek alternative positive coping mechanisms [39].

Similarly, participants reported increased engagement with self-harm or suicide content during dips in their mental health, which were prevalent in this study, as indicated by fluctuations in their well-being measure outcomes over time. These engagements, recalled as “habitual” or “addictive,” highlighted a loss of control during these mental health dips. Previous research has shown a relationship between cognitive inflexibility and addictionlike behaviors [40,41], and a more recent study [42] has indicated that distress-driven impulsivity, in which a person is likely to make rash decisions due to a negative mental state, alongside cognitive rigidity, can lead to addictionlike eating behavior. This emphasizes the potential risk of overreliance on web-based self-harm and suicide content as a coping strategy, particularly during periods of mental health decline, when participants may become more vulnerable to the content they are engaging with. The addictive nature of this behavior also has the potential to negatively impact other important aspects of people’s lives, such as social or occupational functioning [43].

### Disengaging and Reengaging

Key to self-preservation during the web journey was participants’ ability to disengage from web-based spaces. Most participants recorded disengagements in their web journeys in response to life experiences or stressors, such as work stress, bereavement, or a rapid deterioration in mental health. This indicates a resurgence of cognitive flexibility, which reflects previous research showing that individuals become more open to alternative solutions when their perspective is challenged by a significant life event [44]. Although participants demonstrated disengagement attempts from the content during these times, they were usually temporary. This represents a brief state of cognitive flexibility, with reengagement often occurring within days. When disengagement was longer, it tended to coincide with more significant life events such as bereavements, which may indicate more prolonged changes to behavior following extreme circumstances and mental health declines.

Participants also reported that their mental state dictated whether they returned to more helpful or harmful content during the reengagement period. Participants experiencing poorer mental health were more likely to reengage with content they described as “negative” as a type of self-punishment or as a preventative measure against potentially worsening self-harm or suicide behavior. They were also more likely to post their own content, which included help-seeking comments, suicide method inquiries, and “depressive” blog posts. This showed that, although some participants attempted to use their online communities for help during mental health dips, others could find themselves returning to potentially unhelpful or harmful situations. This reflects previous research showing that “active” suicidal ideation is associated with greater cognitive rigidity compared to “passive” suicidal ideation [45]. Often, when reengaging during mental health declines, use would also regress to “addictive” or “habitual” engagements. However, when experiencing mental health improvements during reengagement periods, those who had previously engaged with more “positive” or “recovery-based” content would be more likely to return to this material. This indicates that cognitive rigidity is influenced by mental health state and that, when experiencing mental health changes, participants’ well-being is reliant on earlier web-based encounters with self-harm and suicide content.

### Upskilling Users

Despite this, some participants did experience lasting adaptations to the ways in which they interacted with the content. These more enduring changes were attributable to the skills that participants reported developing in digital efficacy and metacognition. Digital efficacy skills include the ability to use web-based safety mechanisms such as muting, blocking, and self-banning. Participants with digital efficacy skills in the study felt safer and more protected, which acted as a preventative measure against cognitive rigidity. In this study, these participants were likely to be younger, which reflects research showing that digital literacy skills are significantly better in younger cohorts [46]. Despite this, evidence also shows that digital literacy skills can be built over time [47]. This is consistent with the experiences of some participants in this study who reported that their web experiences prompted them to organically develop digital skills and strategies to stay safe over time. This finding has important implications for industry leaders, who should be encouraged to consider ways in which they can empower users by improving accessibility to safety mechanisms on their platforms and sites.

Metacognition skills, or the ability to reflect on one’s own thoughts and behaviors to change one’s responses, were evident in some of the participants [48]. Specific metacognitive abilities such as self-awareness and self-regulation resulted in greater control over their cognitive flexibility. Some described gaining metacognition skills such as self-awareness before their participation in the study, which allowed them to recall changing their responses to content from self-harm behavior to vicarious viewing of material. Others identified metacognitive skills gained through therapeutic input as well as through monitoring web-based behavior and reflecting on it during the study. This may reflect a Hawthorne effect [49] in which participant behavior shifts due to their awareness of being observed in a study. Several diary and ecological momentary assessment interventions have resulted in improved metacognitive skills [50] (Haime, Z, unpublished data, January 2024), and it is possible that metacognition was acquired in this study as a result of completing the research diary. In cases in which participants’ metacognition developed during the study, they noted improvements in their mental health, also indicated by improvements in their well-being outcomes over time. This resulted in the type of self-harm and suicide material they engaged or reengaged with changing from “negative” or “depressive” to “recovery-based” or “positive” in nature. This shows that self-awareness and control while experiencing mental health improvements lead to positive content engagement during periods of cognitive flexibility in this population and has important implications for the development of future target metacognitive interventions.

### Remaining Vulnerable on the Web

However, shifts toward recovery-based content did not necessarily mean that participants were able to fully disengage from their previous self-harm and suicide material. Sometimes, as recovery-based content coexisted alongside more harmful content in web spaces, there was no alternative place in which to access it. On the other hand, some participants expressed a strong connection with the communities they had previously engaged with and reported intentions to remain active in these spaces with a desire to provide support to others. While this altruistic act had benefits, including the ability to continue drawing on support when needed, it left them vulnerable to potentially triggering content. These findings emphasize the strength of web-based self-harm and suicide spaces as a source of comfort and security, which is consistent with previous research on engagement motivators [2,7,10]. Thus, although participants became more aware of the negative outcomes of engaging with web-based self-harm and suicide content and were better able to manage them, the perceived benefits of being involved in a community of like-minded individuals with similar experiences often outweighed the potential costs.

### Limitations

Participants in this study used a diverse range of web-based platforms to access self-harm and suicide content, meaning that attempts to identify patterns in behavior related to the sites used were challenging. However, as common behaviors were observed across participants, it was possible to draw conclusions more broadly about how people engage with web-based self-harm and suicide content over time. Diaries in this study were completed daily by participants, but many had missing entries or were filled out retrospectively. This diluted the advantages of “in-the-moment” diary data capture and resulted in some interview topic guides being less informed by participant data. Despite this, participants reported finding the diaries largely acceptable, and some reported additional benefits to their metacognitive ability related to their completion [51].

While visually observing quantitative data allowed us to identify patterns consistent with participant-reported mental health fluctuations and slight improvements toward the end of the study, our inability to conduct statistical analyses prevented us from identifying any significant differences in participant well-being changes. However, the rich qualitative data and trajectory analysis provided valuable insights into the individual pathways and factors influencing web-based engagement.

In terms of participant characteristics, this study had an underrepresentation of male individuals. Although steps were taken to target male-orientated web spaces for recruitment, uptake remained poor. Furthermore, responses to recruitment were limited, which resulted in possible selection bias and may have affected the representativeness of the sample. In addition, we did not collect data on the educational level or socioeconomic status of the participants involved, limiting our understanding of how demographic characteristics may affect web-based experiences. Half of those recruited at baseline were also lost to follow-up. Strategies were undertaken to limit attrition, including at least 3 attempts to communicate with participants before they were considered lost to follow-up. High attrition rates are consistent with longitudinal studies of self-harm and may represent a selection bias among study completers [52]. Finally, although cognitive flexibility provides a useful framework with which to interpret our findings, it is important to acknowledge that there may be alternative explanations.

### Future Implications

The findings of this study have shown that there are ongoing challenges in navigating the web environment for those engaging with self-harm and suicide content. A key priority for future research should be to establish how engaging with web-based content can be better managed in this population. Consequently, the following should be considered:

1. Inaccessibility to offline support was a significant motivator for participants’ willingness to explore web-based self-harm and suicide–related resources. Therefore, the availability of offline help and support is necessary to limit or moderate initial web-based engagements.
2. This study offers evidence that greater metacognition and digital efficacy can positively influence web-based behavioral control. As individuals are unlikely to completely disengage from web-based content, it is important to prioritize upskilling users. Therefore, interventions should be developed focusing on improving digital literacy and metacognitive skills, such as the diary-based reflections used in this study.
3. A deeper examination of the perceived benefits of web-based engagement is necessary to ensure that these needs can be met in a safer manner both on the web and offline. In addition, it is crucial to critically evaluate the helpfulness of these perceived benefits, such as the impact of “vicarious living” through observing others self-harm.
4. Web-based industry leaders need to produce more tools that empower individuals to regain control of their web-based engagement and improve the safety of web-based spaces where self-harm and suicide content is available. This may include changes to the functions of social media, such as providing further control and management options to users over algorithms and hashtags.

### Conclusions

A balance between cognitive flexibility and rigidity seems necessary to protect individuals when engaging with self-harm and suicide content on the web. While cognitive flexibility may be helpful in certain situations such as exploring new coping strategies, it can also leave individuals vulnerable to harmful content. On the other hand, cognitive rigidity, or the tendency to repeatedly engage with the same type of content, can lead to desensitization, potential impairments in functioning, and an increased severity of harm to oneself. Cognitive rigidity can also prevent people from engaging in harmful behaviors and allow them to consistently engage with content that is helpful and positive. Although life events and changes in mental health state could trigger cognitive flexibility resulting in behavior changes, these were unlikely to become permanent unless participants developed skills such as digital efficacy and metacognition that gave them greater control over their behavior. Despite this, even with improved skills for recognizing and managing web-based risks, individuals still perceived that the benefits of web spaces outweighed the costs, making it difficult to fully disengage.

## Appendix

Multimedia Appendix 1 Supplementary material including data confidentiality statement, additional tables, and descriptive analysis line graphs.

Multimedia Appendix 2 Example safety plan.

## Data Availability

The data sets generated and analyzed during this study are available from the corresponding author on reasonable request.
