# Supplementary material for: The Journey of Engaging With Web-Based Self-Harm and Suicide Content: Longitudinal Qualitative Study
Source: JMIR Infodemiology. 2024 Mar 28;4:e47699. doi: 10.2196/47699 (PMC11009851; doi:10.2196/47699)
Supplement: Multimedia Appendix 1 [file infodemiology_v4i1e47699_app1.docx]

**Supplementary Material**

**Data Confidentiality**

Throughout the study, participants were referred to using identifiers (IDs). IDs (e.g., ID1, ID2, etc) were assigned as participants were sent out study information, to ensure those who participated did not necessarily receive consecutive IDs. These IDs were given to recruited participants, so they knew it and were able to use it to complete the wellbeing surveys. They were also used by transcribers and then researchers during data analysis to ensure familiarity with individual participants’ data, without creating new labels with potential for misinterpretation. For this paper, study IDs have been replaced with new identifiers (e.g., IDA, IDB, etc) to ensure confidentiality of participant information, and that no individuals can be identified by quotes in the text.

**Table S1.***Baseline deductive thematic codes and example quotes.*

| **Themes** | **Example Quotes** |
| --- | --- |
| What?  Types of content and interactions with content | *“Tumblr isn’t a nice place for it. Tumblr, they like to have their self-harm stuff on there.” (IDJ)* |
|  |  |
|  |  |
| When? How? How Often?  Any patterns related to their engagement. | *“There’s been times when I’ve used the online environment an awful lot, particularly around suicidality.” (IDC)* |
|  |  |
|  |  |
| Why?  Motivation and cause (Reasons for engaging) - include mood/mental state | *“I needed to talk about being suicidal without being told, I’m calling the police or an ambulance or you’re going to be put in a psych ward.” (IDH)* |
|  |  |
|  |  |
| Control/Agency | *“sucked into the infinite scrolling.” (IDL)* |
|  |  |
|  |  |
| Impact & Consequences (General) | *“I would feel bad, go on the internet and feel worse.” (IDF)* |
|  |  |
|  |  |
| Impact & Consequences on Self-Harm or Suicide | *“if it’s quite triggering then it could make me feel the same way, like copying behaviours, like doing what they’re doing, I’m thinking of why don’t I do this, so yeah … do you know when teenagers post pictures of self-harm and stuff and I thought oh well I used to do it deeper or I need to do it this way, do you know what I mean, it’s quite competitive.” (IDK)* |
|  |  |
|  |  |
| Being Blocked/Removed/Banned | *“I actually said, ‘This is rubbish’, but you can’t mess with them. They’ll go, ‘You’re going against us so I’m going to get rid of you’, so I thought ‘Okay.’’ (IDG)* |
|  |  |
|  |  |
|  |  |
|  |  |

**Table S2.**

*Participant baseline characteristics for completers (6-month follow-up) vs. non-completers.*

| **Demographic Variables** | **Completers (n=7)**  N | **Non-Completers (n=7)**  N |
| --- | --- | --- |
| **Gender** | | |
| Male | 2 | 2 |
| Female | 5 | 5 |
| **Ethnicity** | | |
| White British | 4 | 3 |
| Asian British | 2 | 0 |
| Black British | 1 | 0 |
| White Other | 0 | 1 |
| Asian Other | 0 | 2 |
| Black Other | 0 | 0 |
| Mixed | 0 | 1 |
| **Age** | | |
| 16-17 | 0 | 1 |
| 18-24 | 3 | 4 |
| 25-35 | 0 | 0 |
| 36-45 | 3 | 1 |
| 46-52 | 1 | 1 |
| 53+ | 0 | 0 |
| **Website/Platform Used to Access Content** * | | |
| Instagram | 1 | 2 |
| Facebook | 5 | 0 |
| TikTok | 0 | 1 |
| Twitter | 2 | 4 |
| Tumblr | 1 | 2 |
| Discord | 0 | 1 |
| WhatsApp | 1 | 0 |
| YouTube | 1 | 2 |
| Suicide Forums | 0 | 3 |

Notes: *participants could choose more than one platform/site

**Measures**

**Generalised Anxiety Disorder Questionnaire (GAD-7)** (Spitzer et al, 2006)

The GAD-7 is typically used to screen for anxiety disorders and consists of seven questions, answered on a four-point scale from ‘Not at all’ to ‘Nearly every day’. Scoring is calculated by assigning scores of 0, 1, 2, and 3 to ‘Not at all’, ‘Several days’, ‘More than half the days’, and ‘Nearly every day’, respectively. Scores for each of the seven questions are then added together to give a total score. The measure has been validated to measure anxiety severity in primary care and the general population (Löwe et al, 2008). Participant score changes over six, monthly timepoints can be viewed in Figure S1.

Figure S1. Line Graph of GAD-7 scores over six, monthly time-points.

**Entrapment Scale Short-Form (ES S-F)** (De Beurs et al, 2020)

The ES S-F is typically used to determine participant feelings of external and internal entrapment from unbearable situations. The measure consists of four questions, answered on a five-point scale from ‘Not at all like me’ to ‘Extremely like me’. Scoring is calculated by assigning scores of 0, 1, 2, 3, and 4 to ‘Not at all like me’, ‘A little bit like me’, ‘Moderately like me’, ‘Quite a bit like me’, and ‘Extremely like me’, respectively. Scores for each of the four questions are then added together to give a total score. The ES S-F was originally validated in a clinical sample of patients following hospital-treated self-harm and a population-based sample of young adults (De Beurs et al, 2020). Participant score changes over six, monthly timepoints can be viewed in Figure S2.

Figure S2. Line Graph of ES-SF scores over six, monthly time-points.

**Patient Health Questionnaire (PHQ-9)** (Kroenke et al, 2001).

The PHQ-9 is typically used as a brief assessment tool to measure severity of depression and consists of nine questions. The measure is answered on a four-point scale from ‘Not at all’, to ‘Nearly every day’. Scoring is calculated by assigning scores of 0, 1, 2, 3 to ‘Not at all’, ‘Several days’, ‘More than half the days’, and ‘Nearly every day’, respectively. Scores for each of the nine questions are then added together to give a total score. The measure was originally validated for the measurement of depression severity in primary care patients (Kroenke et al, 2001). Participant score changes over six, monthly timepoints can be viewed in Figure S3.

Figure S3. Line Graph of PHQ-9 scores over six, monthly time-points.

**Warwick-Edinburgh Mental Wellbeing Scale (WEMWBS)** (Bass et al, 2015).

The WEMWBS is typically used as a general measure of mental well-being, consisting of 14 questions. The measure is answered on a five-point scale from ‘None of the time’ to ‘All of the time’. Scoring is calculated by assigning scores of 1, 2, 3, 4, and 5 to ‘None of the time’, ‘Rarely, ‘Some of the time’, ‘Often’, ‘All of the time’, respectively. Scores for each of the 14 questions are then added together to give a total score. The measure has been validated across samples including the general population, students, adolescents, and secondary mental health care patients (Bass et al, 2015). Participant score changes over six, monthly timepoints can be viewed in Figure S4.

Figure S4 Line Graph of WEMWBS scores over six, monthly time-points.
