# Supplementary material for: The Journey of Engaging With Web-Based Self-Harm and Suicide Content: Longitudinal Qualitative Study
Source: JMIR Infodemiology. 2024 Mar 28;4:e47699. doi: 10.2196/47699 (PMC11009851; doi:10.2196/47699)
Supplement: Multimedia Appendix 2 [file infodemiology_v4i1e47699_app2.docx]

**Keeping you safe during our study**

**DELVE - Understanding engagement with and uses of online self-harm content: a longitudinal qualitative study**

Thank you for joining the DELVE study. We appreciate you taking the time to be involved and share your experiences. Due to the sensitive nature of our research, we have put a few steps in place to look after your safety.

***1) We would like you to fill out the safety plan overleaf and send it to us before we talk for the first time:***

- This plan gives you the chance to identify some self-care options (e.g. listening to music, doing mindfulness exercises, phoning a friend) that help when you are feeling down or anxious, and which you could use if anything we talk about in the research triggers difficult feelings.
- As part of the plan, we would also like you to share with us the name and number of your GP. We will not use this unless we really have to or without talking to you first. However, it is important that we are able to contact someone in the unlikely event that we should become very concerned that you are at imminent risk of serious harm.
- At the end of the plan, we have included some additional sources of support, including mental health helplines and crisis support organisations.

The interviewer will talk through your plan with you before starting the research.

***2) If you feel upset during an interview:***

- There will be chance to pause or stop
- We could arrange for you to talk to a Samaritans volunteer

***3) If you lose connection during the interview:***

We will follow you up to check you are okay and have not disconnected because you are upset.

While it is important that we have these steps in place, it is worth remembering that often people find participating in research interviews to be a positive experience.

**Participant safety plan**

**DELVE - Understanding engagement with and uses of online self-harm content: a longitudinal qualitative study**

My name: ______________________________________________________

My email address: _________________________________________________

My telephone number: ____________________

1. Someone I can contact for support if I feel upset, (e.g. friend, family member, carer, trusted adult)

Name: _____________________________________________________________

1. Name and contact information for my GP:

_____________________________________________________________________

_____________________________________________________________________

(We will only contact your GP if we have serious concerns about your safety and will ask you first).

1. My preferred self-care plan (e.g. a cup of tea and a chat, listening to some music, playing with my pet, mindfulness exercises, talking to a support professional)

______________________________________________________________________

______________________________________________________________________

______________________________________________________________________

______________________________________________________________________

Signature: _______________________________________________________________

Date:

**Below is a list of organisations which could provide additional support and information.**

| **Organisation** | **Support** | **Contacts** |
| --- | --- | --- |
| Samaritans | 24-hour service providing confidential emotional support to anyone in crisis | Helpline: 116 123  Website: [www.samaritans.org](http://www.samaritans.org) |
| SHOUT | Free, confidential, 24/7 text messaging support service for anyone who is struggling to cope. | Text: 85258  Website: www.giveusashout.org |
| Mind | Charity about mental health and related topics. | Helpline: 0300 123 3393  Text: 86463  Website: [www.mind.org.uk](http://www.mind.org.uk) |
| CALM | Charity supporting suicidal men | Helpline: 0800 58 58 58  Website: www.thecalmzone.net |
| HOPELineUK | Specialist telephone helpline to prevent young suicide | Helpline: 0800 068 41 41  Email: [pat@papyrus-uk.org](mailto:pat@papyrus-uk.org?subject=Website%20Contact)  Website: [www.papyrus-uk.org](http://www.papyrus-uk.org) |

If you feel in need of immediate support, please contact Samaritans (116 123) or NHS Choices ([www.nhs.uk/111](http://www.nhsdirect.nhs.uk)) on 111 (both are available 24 hours a day, 365 days a year, and free). Alternatively, please go to, or call, your nearest accident and emergency (A&E) department and tell the staff how you are feeling.
